# Patient satisfaction and sexual issues in vulvar lichen sclerosus treatment: a monocentric certified dysplasia unit survey analysis

**DOI:** 10.1007/s00404-024-07519-w

**Published:** 2024-05-04

**Authors:** Philipp Meyer-Wilmes, Julia Wittenborn, Tomáš Kupec, Rebecca Caspers, Elmar Stickeler, Séverine Iborra

**Affiliations:** 1 Department of Gynecology and Obstetrics, University Hospital of the RWTH Aachen, Pauwelsstraße 30, 52074 Aachen, Germany; 2 Department of Gynecology and Obstetrics, Solingen Municipal Hospital gGmbH, Gotenstraße 1, 42653 Solingen, Germany

**Keywords:** Vulvar lichen sclerosus, Satisfaction, COVID-19 pandemic, Psychologic distress, Sexual function

## Abstract

**Objective:**

Vulvar lichen sclerosus (VLS) is an underestimated chronic disease. It can cause significant symptom burden and sexual dysfunction. This study aimed to evaluate patient satisfaction and current challenges in the management of VLS in a certified dysplasia unit, particularly during the COVID-19 pandemic.

**Methods:**

This survey analyzed patients who had been diagnosed with VLS and treated at our DKG-certified dysplasia unit. The study was conducted during the COVID-19 pandemic in the Department of Gynecology and Obstetrics at the University of Aachen. The questionnaire contained 43 questions on general treatment, diagnostic delays, disease education, psychologic and sexual issues, and specific questions regarding the COVID-19 pandemic. The questionnaires were distributed between January 2021 and September 2023.

**Results:**

This study included 103 patients diagnosed with VLS, who were treated at our certified dysplasia unit. Overall, 48% of the patients were satisfied with the success of the therapy. Most participants reported psychologic problems (36.8%), fear of cancer (53.3%), or sexual restrictions (53.3%). Among the patients, 38% were bothered by the regular application of topical cortisone. However, 72% were willing to undergo treatment for more than 24 months. The COVID-19 outbreak in March 2020 had a significant negative impact on general VLS care from the patient’s perspective (3.83/5 before vs. 3.67/5 after; *p* = 0.046). There was a general request for booklets to inform and educate the patients about their disease. Furthermore, the respondents demanded a telephone hotline to answer the questions and wished for follow-up visits via e-mail to cope better with their current situation.

**Conclusion:**

This study highlights the need for more effective treatments for VLS and an increased awareness of psychologic and sexual distress. To ensure patient well-being and satisfaction, it is imperative to offer individualized care with adequate disease education in a team of specialists from various disciplines.

## What does this study add to the clinical work

**Table:** 

The survey analysis of vulvar lichen sclerosus patients in a DKG-certified dysplasia unit highlights the need for more effective treatments for VLS and increased awareness of psychological and sexual distress.	

## Introduction

Vulvar lichen sclerosus (VLS) is a chronic inflammatory disease of the skin and mucous membranes that is characterized by a lichenoid inflammatory pattern. This inflammatory reaction leads to fibrosis and scarring during the disease and can lead to loss of function [1]. Women and girls commonly report itching, burning pain, and anal or genital bleeding due to fissuring of affected tissues. Women also report painful, less pleasurable, or even impossible sexual intercourse because of stenosis and scarring. Emotional distress and mental health problems can result from symptoms such as itching, pain, tearing, and sexual dysfunction [2]. VLS is typically diagnosed based on its characteristic clinical appearance. In typical cases, biopsy may not be required; however, many clinicians prefer to perform biopsy at presentation. A biopsy should be performed if the clinical diagnosis is uncertain, dysplasia/carcinoma is suspected, or first-line treatment fails. VLS is commonly misdiagnosed as Candida albicans vulvitis or postmenopausal atrophy. This may lead to a diagnostic delay of up to 5 years [3]. Furthermore, missed or delayed diagnoses can lead to disease progression and reduced treatment response [4].

Topical steroids are the recommended first-line treatment, with 60–70% of patients achieving complete remission [5, 6]. Alternatively, topical calcineurin inhibitors and complementary procedures such as photodynamic therapy have been described [7]. Surgical treatment is indicated when stenosis and associated functional limitations occur. In addition to conservative and surgical treatment approaches, the use of lasers for VLS has also been described [8, 9].

Studies have shown that VLS therapy can effectively reduce symptoms. However, the overall patient satisfaction is moderate [5, 10, 11]. One-third of patients with VLS experience a significant reduction in health-related quality of life [10], and some women with VLS may continue to experience sexual dysfunction and less-satisfying sexual activity, even with treatment [12]. Meanwhile, in another gynecological field, an improvement in quality of life and sexual function was shown by surgery with transvaginal bilateral sacrospinous fixation in women affected by second recurrences of vaginal vault prolapse [13].

The coronavirus disease 2019 (COVID-19) pandemic began in December 2019 in Wuhan. Isolation and social distancing dominated interpersonal relations and led to psychologic disorders in many people [14]. Public health organizations have decided to reduce their access to outpatient clinics. In addition, the number of surgical procedures was limited owing to the pandemic. Most patients with chronic diseases have no opportunity for follow-up. Some patients refused to attend follow-up visits due to fear of the pandemic. According to the results of the current research, the COVID-19 pandemic has led to changes in sexual behavior, function, and satisfaction, with several studies indicating an increase in sexual dysfunction and a decrease in sexual activity [15, 16]. Furthermore, access to photodynamic therapy for lichen sclerosus was limited during the COVID-19 pandemic [17].

The primary objective of this patient survey was to evaluate the current state of care in a DKG-certified dysplasia unit. The survey aimed to address concerns, sexual distress, and requirements such as education for effective therapy, with the goal of increasing understanding and improving future therapy. In addition, as the survey was conducted during the COVID-19 pandemic, it aimed to assess the clinical impact of the pandemic on the patients.

## Methods

This survey analysis included patients with vulvar lichen sclerosus who were diagnosed and treated in our DKG-certified dysplasia unit of the Department of Gynecology and Obstetrics at the University Hospital Aachen. A multiple-choice questionnaire with 43 items was developed based on physicians’ long-standing clinical experience, covering general care, treatment satisfaction, diagnostic delay, specific symptoms, and psychologic and sexual issues. Additional questions were asked regarding accessibility, treatment and satisfaction during the COVID-19 pandemic. The questionnaire was distributed to patients attending the consultation at our dysplasia unit and was provided either as a paper version or online in Microsoft Forms. This unit is a referral consultation for particularly difficult cases in which specialists in private practice have the opportunity to obtain a specialized second opinion. Vulvoscopy was performed by experienced and highly qualified AG-CPC-certified personnel. Patients were treated strictly in accordance with the current European guidelines for lichen sclerosus and were also offered a vulvoscopic checkup once a year [18]. This anonymous survey was conducted between January 2021 and September 2023 and was performed in accordance with the principles of the Declaration of Helsinki. Approval was granted by the Ethics Committee of the RWTH Aachen University Faculty of Medicine, Germany in December 2021 (EK 438-21). Informed consent was obtained from all participants. Data extraction and descriptive analyses were performed using SPSS for Macintosh, version 27.0 (IBM Corp., Armonk, NY, USA). Due to the small number of cases, it is not possible to rely on the central limit theorem and assume a normal distribution. Accordingly, the non-parametric inferential statistical hypothesis testing Wilcoxon signed-rank test was used to compare the changes in care after the outbreak of COVID-19 pandemic. Differences were considered statistically significant at *p* < 0.05.

## Results

**Fig. 1:**
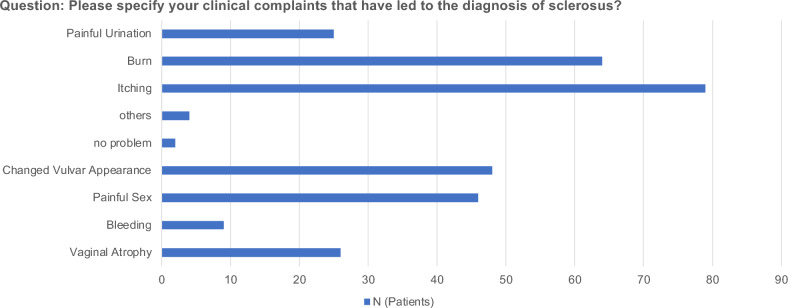
Please specify your clinical complaints that led to a diagnosis of sclerosus

A total of 103 patients participated in this survey. Almost half of the patients (48%) were older than 61 years of age when they completed the survey. Biopsies of the suspicious vulvar regions were part of the diagnosis in 34.0% of the patients, but clinical impressions and symptoms were more common in 45.6% of the patients (Table 1). The most common symptoms were itching (76.7%), burning (62.1%), changes in vulval appearance (46.7%), and pain during sexual intercourse (44.7%) (Fig. 1).

**Table 1:** Patient characteristics

Characteristics	*N* (%)
Age (years)	
18–30	8 (7.7)
31–41	9 (8.7)
42–50	11 (10.7)
51–60	24 (23.3)
> 61	49 (47.6)
Type of VLS diagnosis	
Biopsy	35 (34.0)
Clinical	47 (45.6)
Preliminary suspected diagnosis	21 (20.4)
Time from the beginning of symptoms to presentation to the doctor (months)	
Directly	36 (35.0)
3–6	29 (28.2)
6–12	8 (7.8)
12–24	4 (3.9)
> 24	24 (23.3)
Missing	2 (1.9)

*VLS* vulvar lichen sclerosus

**Fig. 2:**
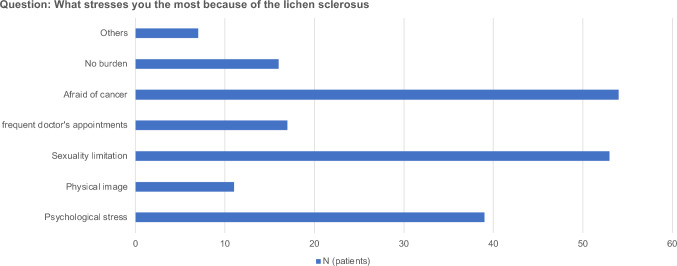
What stresses you most because of lichen sclerosus?

A total of 48.0% of patients were satisfied with the success of the therapy and the majority of respondents reported having psychologic problems (36.8%), fear of cancer (53.3%), or sexual restrictions (53.3%). Among patients who underwent VLS treatment, 75.7% were expected to avoid cancer, whereas 58.3% were expected to have an increased chance of being cured (Fig. 2). In addition, 48.5% of the patients were expected to improve their quality of life through VLS treatment.

**Fig. 3:**
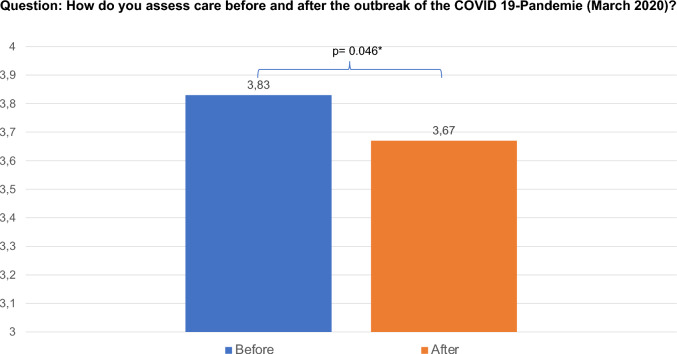
Assessment of care before and after the outbreak of the COVID-19 pandemic. 1 = bad, 5 = very good

Regarding their disease and previous therapy, 61.2% of patients considered it difficult to manage their fear of cancer. Overall, 65.0% of patients felt involved in their treatment decisions. In one-fourth of the patients (25.0%), it took more than 24 months from the beginning of the typical lichenoid symptoms to the patient’s presentation to the doctor and likewise from the presentation to the doctor until the correct diagnosis was obtained. Referral to the certified dysplasia unit was faster, with only 11.0% of patients waiting for more than 24 months, and 39.0% referred immediately after diagnosis. Of the patients, 21.3% had comorbidities such as arterial hypertension, 34.0% had thyroid disease, and 39.9% had no comorbidities. From the patients’ perspective, the main causes of the disease were stress (39.8%) and disorders of the immune system (33.8%). Respondents indicated that the outbreak of the COVID-19 pandemic in March 2020 had a significant negative impact on the general care of VLS. Before the pandemic, the average satisfaction with care was 3.83/5; after the outbreak, it was 3.67/5 (*p* = 0.046; Fig. 3). However, the COVID-19 pandemic has not negatively affected access to physicians, confidence, or psychologic distress.

**Fig. 4:**
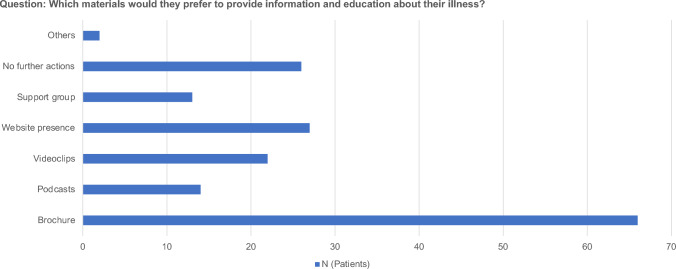
Question: Which materials would they prefer to provide information and education about their illness?

More than one-third 38.0% of the patients were bothered by the regular application of topical cortisone; however, 72.0% were willing to be treated for more than 24 months. To favorably influence the progression of their illness, 76.7% of the participants followed doctors’instructions, 41.8% tried to think positively, and 31.1% lived consciously and took time for themselves. There was a general request for booklets to inform and educate the patients about their disease (Fig. 4). Respondents requested a telephone hotline to answer disease-specific questions and wished for follow-up visits via e-mail to better cope with their current situation.

## Discussion

VLS is a chronic disease that affects women in varying degrees throughout their lives. It is associated with varying levels of satisfaction and psychologic and sexual impairment. In general, patients diagnosed with lichen sclerosus reported a moderate level of satisfaction with their treatment, especially in terms of symptom alleviation. Our study showed that half of the patients (48.0%) were satisfied with the success of therapy. A cross-sectional study revealed that despite undergoing treatment, approximately one-third of patients with LS experience a significant decline in their health-related quality of life (HRQoL) [19]. According to our analysis of 25.0% of patients, it took more than 24 months from the beginning of the typical lichenoid symptoms to the patient’s presentation to the doctor and likewise from the patient’s presentation to the doctor until the correct diagnosis was obtained. In contrast, referrals to our certified dysplasia unit were faster. Indeed, 39% of patients were diagnosed immediately by a specialist, whereas only 11.0% waited for more than 24 months. Delays in diagnosing VLS can lead to complications such as scarring or cancer development [20]. Faster access to physicians specializing in VLS care reduces diagnostic delays and enables patients to comprehend and manage their conditions more effectively, leading to improved compliance.

An important aspect of managing chronic genital diseases is addressing their impact on mental health. Patients often experience anxiety, depression, and stress associated with their disease, which can adversely affect their quality of life and treatment outcome. VLS has been shown to have a significant impact on the mental and psychosocial health of women [21, 22]. In our study, 61.2% of patients considered it difficult to manage their fear of cancer. The most common symptoms were itching (76.7%), burning (62.1%), changes in the vulvar appearance (46.7%), and pain during sexual intercourse (44.7%). More than half of patients experienced psychologic distress and sexual restrictions. The correlation between the negative influence of vulvar lichen sclerosus on female genital self-image and sexual arousal, orgasm, and satisfaction rate is concerning. Individuals also report a significantly lower frequency of sexual activity, lower levels of satisfaction with sexual activity, depression, and poor quality of life [12]. More than half of the patients in our study experienced psychologic distress, fear of cancer, or sexual restrictions. Patients with these conditions have significant advantages in terms of interdisciplinary care and therapy tailored to their individual needs.

Enhancing patient education on vulvar lichen sclerosis is crucial as it empowers patients to actively manage their condition. Education can help them understand their symptoms, identify triggers, and make informed decisions regarding treatment options. For instance, informing patients about the possible adverse effects of medications can assist them in making informed decisions regarding their treatment plans and managing any negative consequences that may occur [23]. According to a study on endometriosis patients, educating women about their condition and providing them with easily accessible information can improve treatment adherence and quality of life [24]. Overall, improving education on vulvar lichen sclerosis can empower patients to take control of their health and enhance their quality of life.

Owing to the COVID-19 pandemic, mental health issues have arisen and there have been great societal concerns regarding fewer care options for nononcological diseases [25]. Continuous stress during the COVID-19 pandemic may increase the risk of disease progression. During the COVID-19 pandemic, the treatment algorithms for vulvar cancer have been modified. VIN 2 and 3 qualifying for resection can be performed with a delay of 10–12 weeks [26]. An increased risk of developing squamous cell carcinoma in VLS has been described [27, 28]. One study revealed that the 20-year risk of squamous cell carcinoma in women with VLS was 6.7% [29]. Follow-up visits are necessary to monitor vulvar LS progression. From the patient’s perspective, our analysis showed that the COVID-19 outbreak had a significant negative impact on the overall care of patients (3.83/5 before vs. 3.67/5 after; *p* = 0.046). Access to VLS specialists, confidence in physicians and psychologic distress have remained stable during the COVID-19 pandemic. This might be due to the well-structured patient care in our certified dysplasia unit even before the outbreak of the pandemic.

This study has several limitations, including its retrospective design and small number of enrolled patients. Considering the rarity of VLS and the limited observational period, particularly during the COVID-19 pandemic, the number of patients enrolled in this study is noteworthy. The questionnaire used in this study should be interpreted without validation in a larger population. However, there are no comparable reports in the literature on patient satisfaction and sexual issues in such a large population. The single-center focus on a highly specialized dysplasia unit, which may have led to potential selection bias, is another limitation of this study. On the other hand, the analysis provides a comprehensive overview of treatment and its challenges in a large specialized and certified center in the region. Future studies should include primary and secondary care centers in their analyses to better reflect the current landscape of care across all levels and to provide a more comprehensive understanding of VLS. Furthermore, future research should take advantage of the great potential of multidisciplinary care and post-COVID-19 telemedicine opportunities in integrative patient care.

In conclusion, this survey highlights the need to improve patient satisfaction with successful VLS treatments. The survey respondents acknowledged that healthcare providers should provide more information on their websites and brochures. They also agreed that there should be more emphasis on sexual distress. Individualized care and multidisciplinary teams are essential to address each patient’s unique needs.

## Data Availability

The dataset generated in this study are available from the corresponding author upon request.
